# Motor Unit Action Potential Clustering—Theoretical Consideration for Muscle Activation during a Motor Task

**DOI:** 10.3389/fnhum.2018.00015

**Published:** 2018-01-31

**Authors:** Michael J. Asmussen, Vinzenz von Tscharner, Benno M. Nigg

**Affiliations:** Faculty of Kinesiology, University of Calgary, Calgary, AB, Canada

**Keywords:** motor units, EMG control, neural control of movement, fatigue, EMG signal processing, motor unit recruitment, synchronization, synchronized oscillations

## Abstract

During dynamic or sustained isometric contractions, bursts of muscle activity appear in the electromyography (EMG) signal. Theoretically, these bursts of activity likely occur because motor units are constrained to fire temporally close to one another and thus the impulses are “clustered” with short delays to elicit bursts of muscle activity. The purpose of this study was to investigate whether a sequence comprised of “clustered” motor unit action potentials (MUAP) can explain spectral and amplitude changes of the EMG during a simulated motor task. This question would be difficult to answer experimentally and thus, required a model to study this type of muscle activation pattern. To this end, we modeled two EMG signals, whereby a single MUAP was either convolved with a randomly distributed impulse train (EMG-rand) or a “clustered” sequence of impulses (EMG-clust). The clustering occurred in windows lasting 5–100 ms. A final mixed signal of EMG-clust and EMG-rand, with ratios (1:1–1:10), was also modeled. A ratio of 1:1 would indicate that 50% of MUAP were randomly distributed, while 50% of “clustered” MUAP occurred in a given time window (5–100 ms). The results of the model showed that clustering MUAP caused a downshift in the mean power frequency (i.e., ~30 Hz) with the largest shift occurring with a cluster window of 10 ms. The mean frequency shift was largest when the ratio of EMG-clust to EMG-rand was high. Further, the clustering of MUAP also caused a substantial increase in the amplitude of the EMG signal. This model potentially explains an activation pattern that changes the EMG spectra during a motor task and thus, a potential activation pattern of muscles observed experimentally. Changes in EMG measurements during fatiguing conditions are typically attributed to slowing of conduction velocity but could, per this model, also result from changes of the clustering of MUAP. From a clinical standpoint, this type of muscle activation pattern might help describe the pathological movement issues in people with Parkinson’s disease or essential tremor. Based on our model, researchers moving forward should consider how MUAP clustering influences EMG spectral and amplitude measurements and how these changes influence movements.

## Introduction

During dynamic and fatiguing contractions to volitional failure, bursts of muscle activation appear in the electromyography (EMG) signal. This activation of the muscle has been observed in frequencies anywhere from 20 Hz to 60 Hz and these bursts of activation are observed both in surface and indwelling EMG signals. From a theoretical standpoint, this type of muscle activation of the muscle is likely due to motor unit action potentials (MUAP) firing temporally close to one another. Although there are invasive and non-invasive experimental methods to record a proportion of motor unit activity (De Luca et al., 2006; Negro et al., 2016), it is often difficult to determine the precise firing patterns of all motor units in a whole muscle. Using surface EMG, further difficulties arise because of distorted signals being recorded due to volume conductor effects, amplitude cancellation, or cross-talk (Solomonow et al., 1994; Winter et al., 1994; Farina et al., 2004b; Keenan et al., 2005, 2006; von Tscharner, 2010; De Luca et al., 2012; Mesin, 2013). Since these distortion effects are present in experimental time series EMG, researchers often choose to model the EMG signal to understand the underlying sequence of activation to create a muscle activity pattern, while controlling for factors such as location and orientation of muscle fibers, motor unit recruitment patterns, rate coding, conduction velocities and/or shapes of MUAP and how these factors contribute to an EMG signal (Fuglevand et al., 1993a; Farina and Merletti, 2000; Stegeman et al., 2000; Farina et al., 2002).

During fatiguing conditions, there are a number of changes that occur in the EMG signal. In a time-series of EMG data during fatiguing contractions, there is an increase in the amplitude of the EMG signal and this increase is typically associated with an increased recruitment of motor units. In the frequency domain, the mean and median frequency of the power spectrum shifts to lower frequencies during a fatiguing contraction, which has been attributed to: (a) an increase in the width and shape of the MUAP due to slowing of conduction velocities; and/or (b) due to synchronous firing of motor units (Bigland-Ritchie, 1981; Merletti and Lo Conte, 1997; Dimitrova and Dimitrov, 2003; Gandevia, 2008). The usual downshift in mean or median frequency observed during isometric contractions, however, is often not observed in a consistent manner across experimental studies and conduction velocity does not fully explain the reduction in mean and median frequency (Dimitrova and Dimitrov, 2003). Therefore, there must be other factors that strongly influence EMG signal during fatiguing contractions such as the motor unit synchronization (Yao et al., 2000).

These changes in the EMG power spectrum are not only restricted to fatiguing contractions. Certain clinical populations also present with distinct changes in EMG and the resulting movement. Particularly, people with Parkinson’s disease and essential tremor have a movement disorder of periodic or oscillatory movements of the limbs. These periodic movements are likely driven by an altered muscle activation pattern and in fact, people with these disorders also show changes in the EMG time-series signal and power spectrum marked by the low frequency peak compared to individuals without these disorders (Rossi et al., 1996; Chen et al., 1997). Further, a burst like firing pattern of motor units also becomes evident in the surface EMG signal with the presence of a vibratory stimulus, suggesting that this type of motor unit firing could be driven by afferent feedback (Lebedev and Polyakov, 1992). Given that bursts of muscle activity occur during both isometric and dynamic fatiguing contractions, clinical populations and artificial sensory input (Piper, 1912; Hagbarth et al., 1983; Lebedev and Polyakov, 1992; Rossi et al., 1996; Chen et al., 1997; von Tscharner et al., 2011; Maurer et al., 2013) and we hypothesize that these short bursts of muscle activity are due to motor units being activated temporally close to one another, we believe that using an EMG model that controls the firing patterns of muscles could explain the EMG changes observed during these conditions.

We define motor units that fire in close proximity to one another (i.e., 5–100 ms) as motor unit action potential “clustering”. Typically, motor units that fire in close proximity to one another have been referred to as synchronization. This synchronization has been defined as either short-term or long-term synchronization (De Luca et al., 1993). Short-term synchronization is defined as motor units that fire within ~5 ms of each other, while long-term synchronization is defined as motor units that fire anywhere between ~6 ms and ~80 ms. A large amount of research has focused on short-term motor unit synchronization, but less attention has been given to long-term synchronization. Our MUAP clustering is similar, but not the same as long-term synchronization such that we defined clustering as MUAP that fire within 5–100 ms. It is important to note that MUAP clustering does not indicate that the same motor units are required to fire within consecutive clusters and that the clustering of motor units could be comprised of different motor units across different clusters, which is different from De Luca et al. (1993) definition of long-term synchronization. Although it may appear that long-term synchronization is similar to clustering, there is value of introducing the concept of clustering vs. long-term synchronization. With a model, we can precisely control the degree of MUAP clustering and determine the effects of MUAP clustering on a modeled EMG signal, which is different than creating a signal due to long-term synchronization. Previous work by Yao et al. (2000) has provided a thorough investigation of how short-term synchronization affects EMG and force characteristics such as amplitude and spectral measures. Although the present study is somewhat similar to Yao et al. (2000), these researchers have not investigated the effects of motor units that are activated in clusters (i.e., 5–100 ms window). We suspect that this MUAP clustering will help determine the unexplained mechanisms of EMG changes during dynamic and fatiguing contractions as well as potentially the EMG changes in Parkinson’s disease and essential tremor.

The purpose of this study is to increase the understanding of the effect of motor unit action potential clustering (MUAP arriving between >5 ms and <100 ms) on EMG power spectra using a simplified model of an EMG signal. The first hypothesis is that the EMG time and frequency domain analyses (i.e., mean and median frequency, shape of the power spectrum) observed during fatiguing conditions and certain clinical conditions can be explained by a modeled EMG signal including clustering of motor units during a motor task. The second hypothesis is that, even if there is a larger amount of random superposition of MUAP, a small amount of clustering will still yield distinctive, observable changes in the EMG time and frequency domain. The proposed model can be used to explain the phenomenon of power spectra changes seen in previous work and our sequence of activation during a motor task could provide novel insight to the activation of muscles during dynamic tasks, fatiguing contractions and clinical conditions that to date, has been unaccounted for by previous research.

## Materials and Methods

### EMG Model

**Figure 1:**
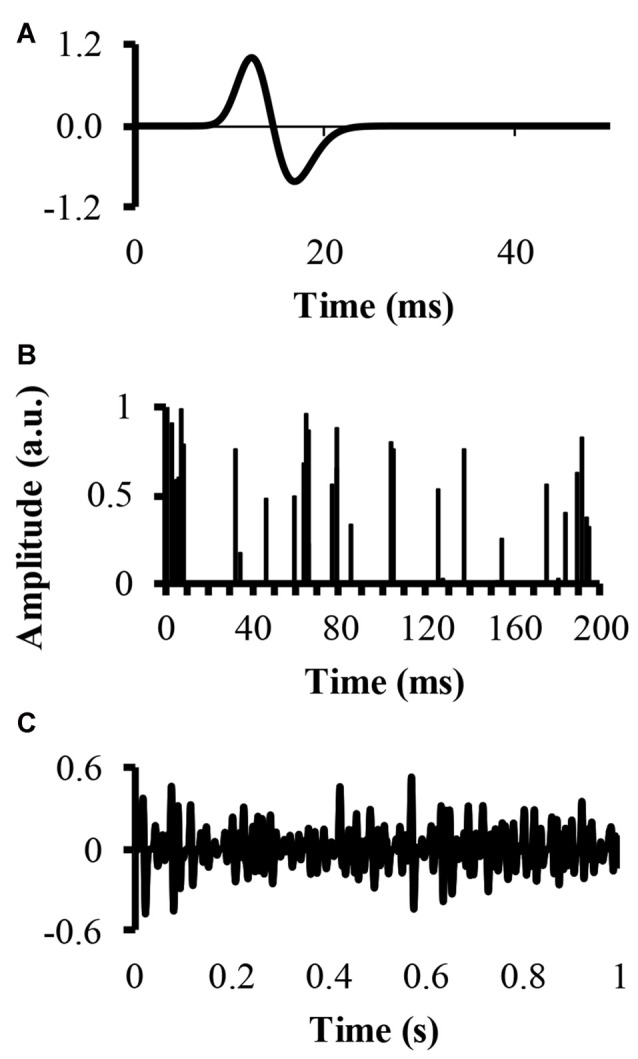
Simulation of an electromyography (EMG) signal created from clusters of motor unit action potentials (MUAP). **(A)** The top figure displays the modeled single MUAP. **(B)** The middle figure displays the clustered impulse train representing a train of firing instants. **(C)** The bottom figure shows the simulated clustered EMG signal as a result of the convolution.

An EMG signal, called EMG-rand, of 54.6 s in time duration, corresponding to 2^17^ points recorded with a sampling rate of 2400 samples, was simulated. The simulation convolved a single MUAP with a set amount of randomly located impulses in time, resulting in a number of randomly distributed MUAP of varying amplitude. The modeled single MUAP was a close replicate of the one published by (Farina et al., 2004a; Figure 1A). A second EMG signal, called EMG-clust, was simulated by dispersing a number of clustered motor units at random locations within a time window of 54.6 s. The clustered motor units occurred at a number of random start locations (start-loc) and a number of additional impulses of random amplitudes between 0 and 1 (add-imp) were defined further in time of each start-loc (start-loc = 500 different locations) within a time interval called cluster duration. At these start-loc, additional, randomly distributed impulses (add-imp = 25 impulses) were added in windows lasting for different durations (defined below) giving a total number of impulses to be 12,500. The modeled EMG-clust was obtained by convolving the add-imp with the same single MUAP (Figure 1B). The result of this convolution was an artificial EMG signal of randomly distributed impulses within a defined cluster-duration window, yielding an EMG signal consisting of clustered MUAP. The function of varying the amplitude of the impulses was to create MUAP of varying amplitudes as a result of the convolution that could represent smaller and larger motor units.

In real experimentally obtained EMG signals, clusters of MUAPs and randomly distributed MUAP may most likely be “mixed” and not comprised of only clustered MUAP. In the context of this model, an EMG mixed signal could contain both randomly distributed and clustered MUAP. A third EMG, called EMG-mixed, was simulated by forming a sequence of impulses containing a fixed fraction of clustered MUAP. For example, an EMG-mixed could be obtained by 50% randomly distributed MUAP and 50% clustered MUAP. For all of these three modeled signals, the number of active motor units was kept constant. This constraint was achieved by making the number of impulses that were convolved with the single MUAP equal across all three modeled signals. Thus, this constraint would remove any effects of additional motor unit recruitment on the modeled EMG signal.

### Model Parameters

**Figure 2:**
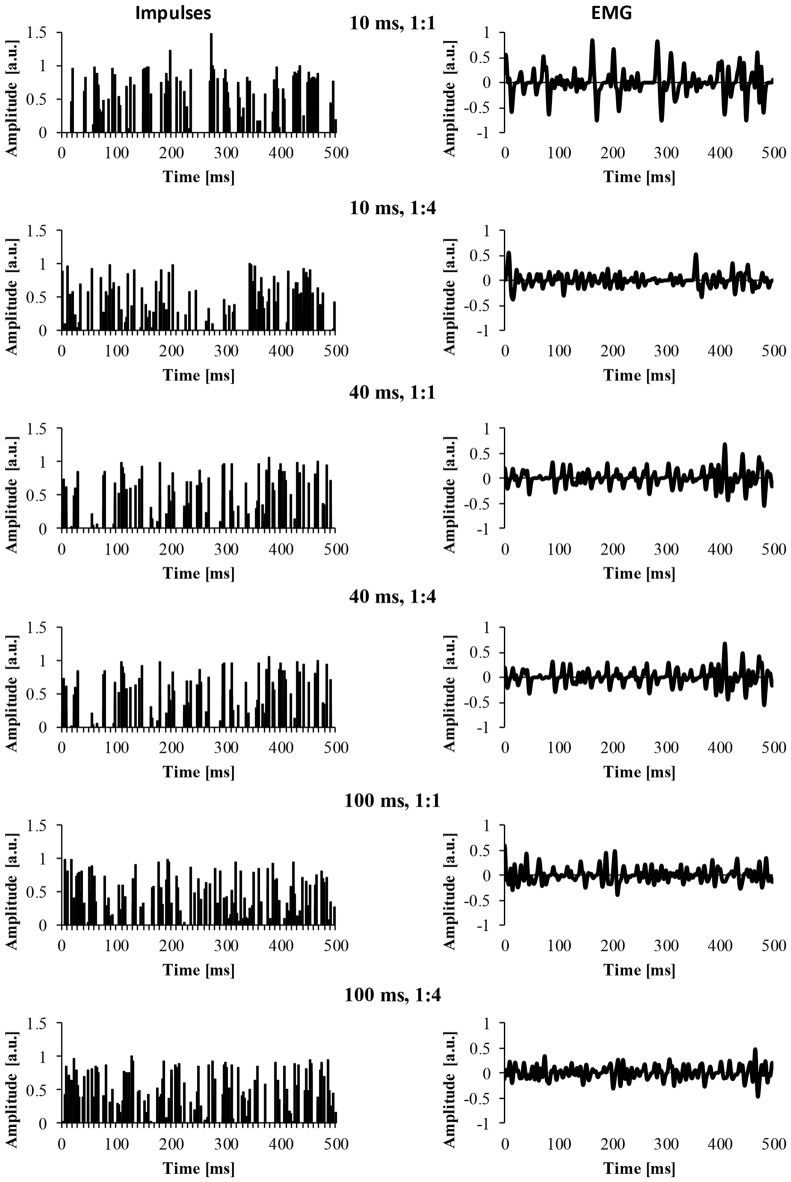
The impulses and resulting EMG signal due to different model parameters. The left column represents the impulse signal over 500 ms. The right column represents the EMG signal due to the convolution of the impulses and the MUAP. The title of each row indicates the cluster duration (e.g., 10 ms) followed by the cluster ratio (e.g., 1:1).

For the EMG clustered and EMG mixed signal, we also modified a number of parameters of the EMG signal. The first parameter was the cluster-duration, which defined the window size that the additional impulses were constrained to fire in. For example, if the cluster-duration is 40 ms, the additional impulses (i.e., 25 impulses) are constrained to fire within the 40 ms window. We created EMG signals of different cluster-durations (5–100 ms) in 5 ms increments, yielding 21 different cluster durations. Additionally, it is likely that MUAP clustering is not a phenomenon that is all or nothing and instead, it’s likely that there are different degrees of clustering. Thus, another parameter that was added to the model was the ratio of clustered to randomly distributed MUAP. Specifically, we modeled EMG signals with five different cluster ratios (i.e., ALL, 1:1, 1:2, 1:4, 1:10) to determine how the degree of clustering influences the EMG signal. A ratio of 1:1 would indicate that 50% of MUAP are clustered while 50% are randomly distributed, a ratio of 1:2 would indicate that 33% of MUAP were clustered, a ratio of 1:4 would indicate that 20% of MUAP were clustered, and a ratio of 1:10 would indicate that 10% of MUAP were clustered. Figure 2 shows example signals of the impulses and the resulting EMG due to different cluster durations (10, 40 and 100 ms) for two different cluster ratios (1:1, 1:4).

### Frequency Domain Analysis

A Fourier transformation was performed on each modeled EMG to obtain its power spectral density. The power spectrum was computed using sequential Fourier transformations. A Fourier transformation was completed in sequences of 2^10^ points and the sequential power spectral densities were averaged, yielding a frequency resolution of 2.34 Hz (Rosenberg et al., 1989). There was no overlap of the windows for the Fourier transformation. Although this process of sequential Fourier transformations prevents the requirement of much further filtering, an additional triangular moving average (convolution with a vector [1/4, 2/4, 1/4]) was used to smooth the power spectra for presentation purposes in the figures. To control for spectra containing more total power than another signal, all power spectra were normalized by dividing the spectra by the total power of the signal, providing a normalized power spectral density (nPSD). For example, one EMG signal may have more amplitude cancellation compared to another EMG signal and therefore, the signals were normalized. From each modeled nPSD, the mean and median frequencies were computed (Farina and Merletti, 2000; Winter, 2009). Below, all power spectra will represent the normalized values.

### Time-Frequency Domain Analysis

A wavelet analysis was used to resolve the power in 13 frequency bands of each modeled EMG signal as performed elsewhere in the literature (von Tscharner, 2000). The power was summed across all frequency bands to extract the total power of the signal across the entire modeled EMG signal in time. The total power of the EMG signal was then averaged across the entire 54.6 s period to determine the mean EMG power of each EMG signal. This analysis was performed for each EMG signal created from the 21 different cluster durations and the five different cluster ratios.

### Statistical Analysis

To determine if there were significant differences for the dependent variables measured, the model simulation was performed with 100 iterations. After the iterations, a two-way repeated measures analysis of variance (ANOVA) with factors CLUSTERRATIO (five levels: all, 1:1, 1:2, 1:4, 1:10) and CLUSTERDURATION (21 levels: 5–100 ms in 5 ms increments). If there was a significant interaction effect, the location of the significant differences was determined with paired comparisons. If the data did not meet the assumptions of the parametric ANOVA, a Greenhouse-Geisser correction was implemented.

## Results

### Frequency Domain Analysis

**Figure 3:**
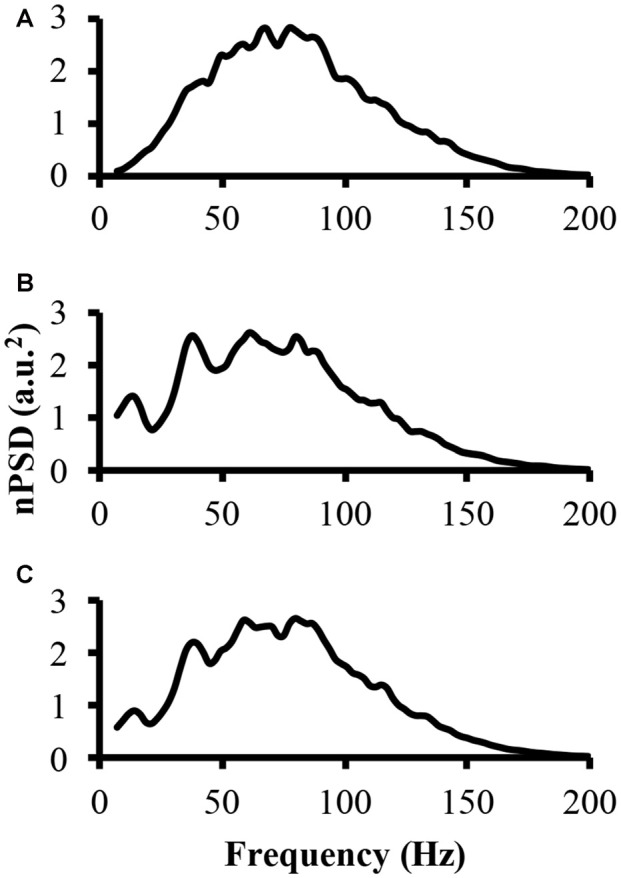
The power spectrum of the three modeled EMG signals. **(A)** The top figure is the power spectrum of the EMG signal created from randomly distributed MUAP. **(B)** The middle figure is the power spectrum of the EMG signal created from the clustered MUAP (i.e., cluster-duration = 40 ms). **(C)** The bottom figure is the power spectrum of an EMG signal created from 50% clustered MUAP and 50% randomly distributed MUAP.

To address part of the first hypothesis that EMG frequency domain changes as a result of clustering, we examined the power spectrum from the different modeled EMG signals. To start, the power spectrum was determined for the EMG-rand signal. The nPSD of this EMG-rand signal shows a bell shaped distribution (Figure 3A). The power spectrum of the EMG-clust signal was quite distinct compared to the EMG-rand signal. Figure 3B shows the power spectrum of the EMG-clust signal when the cluster duration was 40 ms and the signal was comprised of 50% clustered MUAP (i.e., 1:1). The results indicate that MUAP clustering causes: (1) an additional relative power added to the nPSD at frequencies below 25 Hz; and (2) structure to the power spectrum such that there are peaks or oscillations added in comparison to the relatively smooth bell-shape of the EMG-rand nPSD.

The nPSD of the EMG-clust showed distinct differences in the shape of the EMG-rand nPSD. Specifically, clustering leads to a bimodal or even multimodal nPSD distribution (Figure 3B). The first main peak, in relation to the lowest frequency bin, of the nPSD of the EMG-clust signal (i.e., Figure 3B) shifted to lower frequencies compared to the main peak nPSD of the EMG-rand signal (i.e., Figure 3A). One also has to notice that spectral power is observed down to very low frequencies, frequencies <20 Hz. Therefore, the clustered MUAPs are able to create very prevalent changes in the EMG power spectrum distribution in relation to an EMG-rand signal.

**Figure 4:**
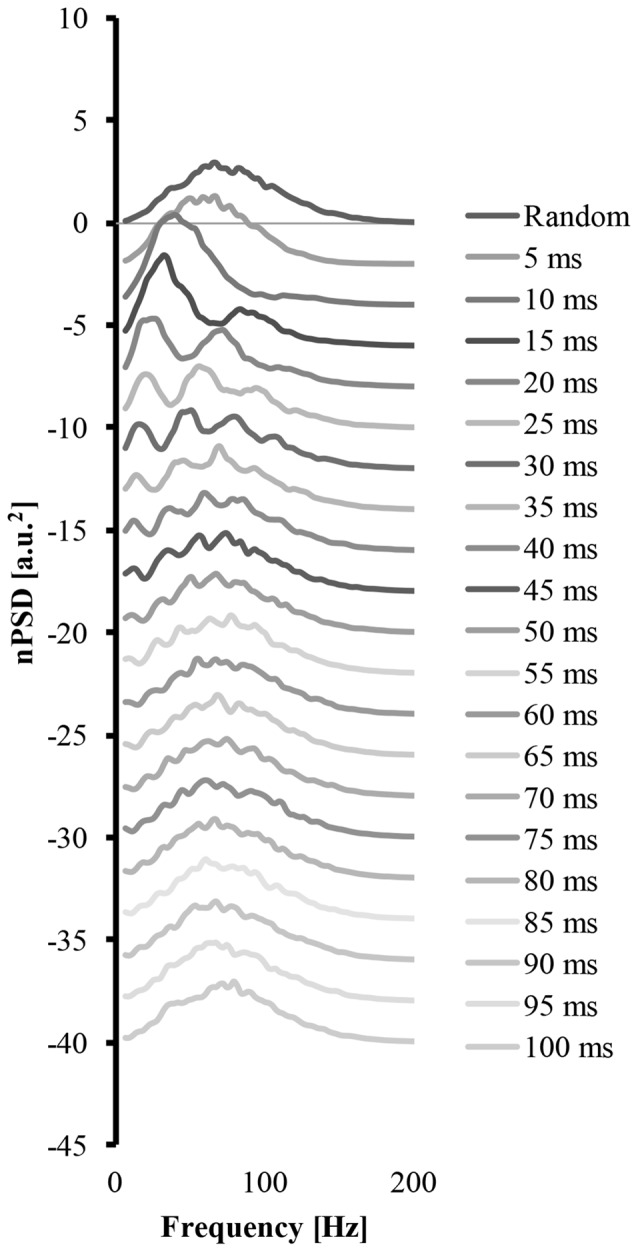
The power spectrums of an EMG signal created from clustered MUAP with varying cluster-duration. The top line is the power spectrum created from an EMG random signal. Each consecutive descending line contains the power spectrum from an EMG clustered signal starting at a 5 ms cluster duration and increasing the cluster-duration by 5 ms with each line.

Figure 4 shows how a systematic increase of the cluster-duration from 5 ms to 100 ms, while keeping the start-loc and add-imp constant at 500 and 25 (arbitrary units), respectively, reveals how the cluster-duration gradually shifts the peaks in the power spectrum to different frequencies and changes the shape of said power spectrum (Figure 4). These power spectrums were produced with the EMG signal created from “ALL” clustered MUAP. If the cluster-duration is very small, the clustered MUAP would likely be nearly superimposed, and therefore the MUs would appear to get activated by a common input. The corresponding EMG-mixed nPSD of a small cluster-duration window (Figure 4 second line, cluster-duration = 5 ms) has a similar shape of the EMG-rand nPSD (compare Figures 4, 3A) because the small window size would not cause a long burst in the EMG signal. As a result, a very small window size for clustering is very similar to the impulses occurring in the EMG random signal. On the other extreme, the shape of the nPSD comprised of a very large cluster-duration (i.e., 100 ms, Figure 4, bottom line) also corresponds to the power spectrum of randomly distributed MUAPs. The effects of cluster-duration are more prevalent with durations ranging from 5 ms to 40 ms. As the cluster-duration increases (i.e., Figure 4 descending from top to bottom power spectrum), two peaks emerge in the power spectrum. One peak in the nPSD shifts to a lower frequency and gradually a second peak emerges at relatively higher frequency of ~100 Hz (Figure 4, 4th line). Ultimately, as the cluster duration continues to increase these peaks begin to disappear.

**Figure 5:**
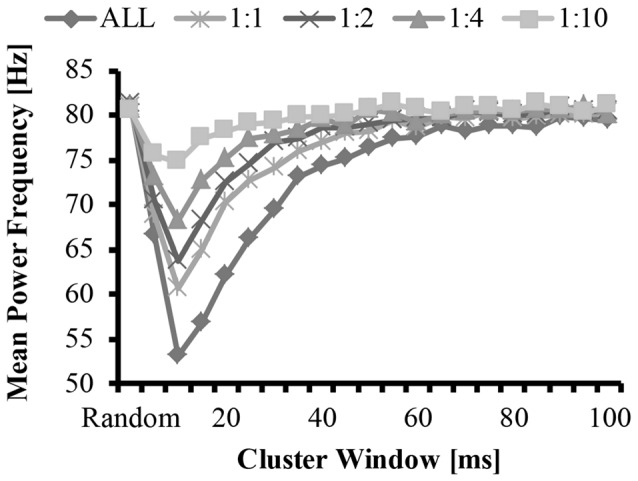
Mean frequency as a function of cluster-duration window size for a representative iteration. The diamonds represents an EMG signal with all clustered MUAP. The circles represent the mean frequency of an EMG signal with a 1:1 ratio of clustered to randomly distributed MUAP. The asterisks represent the mean frequency of an EMG signal with a 1:2 ratio of clustered to randomly distributed MUAP. The triangles represent the mean frequency of an EMG signal with a 1:4 ratio of clustered to randomly distributed MUAP. The squares represent the mean frequency of an EMG signal with a 1:10 ratio of clustered to randomly distributed MUAP.

The bimodal distribution of the EMG-clust nPSD likely causes a substantial shift in the mean and median frequency of the spectrum (Figure 5, details explained below). Therefore, the mean and median frequency was calculated for the nPSD of the each modeled EMG-clust signal with the cluster-duration ranging from 5 ms to 100 ms. This figure also contains the metrics of the power spectrum contained in Figures 2, 3. The cluster-duration, therefore, has a mixed effect on the mean frequency. With a very small cluster-durations (i.e., 5 ms) there is minimal to no change in the mean frequencies in comparison to the mean frequency of the EMG-rand nPSD (fmean = 80 Hz; Figure 5). As the cluster duration increases, there is a decrease in the mean frequency of the power spectrum with a minimum at a cluster duration of 10 ms (i.e., fmean = 53.62 Hz). From this trough, as the cluster duration increases, there is a gradual, relative increase in the mean frequency. This increase continues until the mean frequency matches that of an EMG-rand nPSD at a cluster duration of 65 ms. Thus, a power spectrum with clustered MUAP, compared to randomly distributed MUAP, causes the mean frequency to decrease. It could be argued that the changes in the EMG model may only affect the one power spectrum metric used in previous studies such as mean frequency, but our results indicated that the same change occurs in the median frequency.

These effects of MUAP clustering were very prominent as the EMG-clust only contained MUAP arriving temporally close to one another (i.e., ALL). To answer part of the second hypothesis, we modulated the ratio of clustered to randomly distributed MUAP. The nPSD of the different cluster ratios making up the EMG-mixed signal still reveals how MUAP clustering influences its structure (Figure 3C) with effects that are not as prominent. This varying ratio might also result in a damping of the magnitude of the nPSD peaks (Figure 4) and impact the metrics of the power spectrum. Thus, there could be two parameters that can alter the mean frequency of the power spectrum, the cluster duration, as presented above, and the cluster ratio (i.e., 1:1, 1:2, 1:4, or 1:10). The variation of the ratio of clustered to random MUAP showed different effects on the mean frequency such that it reduced the absolute decrease in mean frequency and this effect was more pronounced with a smaller number of clustered MUAP compared to randomly distributed MUAP. For example, the decrease in mean frequency was larger for the 1:1 ratio compared to the 1:10 ratio. Even though the magnitude of the mean frequency decrease was reduced with varying ratios, it did not dramatically change the cluster-window duration, where the lowest mean frequency occurred (i.e., Figure 5, cluster-duration = 10 ms).

The statistical analysis of the mean EMG power as a function of cluster ratio and cluster duration revealed that there was a significant interaction effect (*F*_(41.7,4131.6)_ = 4199.5, *p* < 0.001). From the significant interaction effect, the important comparisons are the differences in mean frequency between the EMG signals created from different cluster ratios for a given cluster duration (e.g., comparison across five signals with a cluster duration of 10 ms) as well as the comparison of the mean frequency of the EMG-rand signal in comparison to an EMG signal containing clustered MUAP (e.g., 1:1 cluster ratio) for a given cluster duration (e.g., 10 ms). For the comparisons across the different cluster ratios, every comparison was significantly different (*p* < 0.05), except for the following comparisons: 1:2 vs. 1:4 cluster duration = 85 ms, 1:4 vs. 1:10 cluster duration = 90 ms. For the comparisons of the EMG-rand to the clustered EMG signal, the mean frequency was significantly different for each clustered EMG signal in comparison to the EMG-rand signal when the cluster duration was 5–40 ms.

### Time-Frequency Domain Analysis

**Figure 6:**
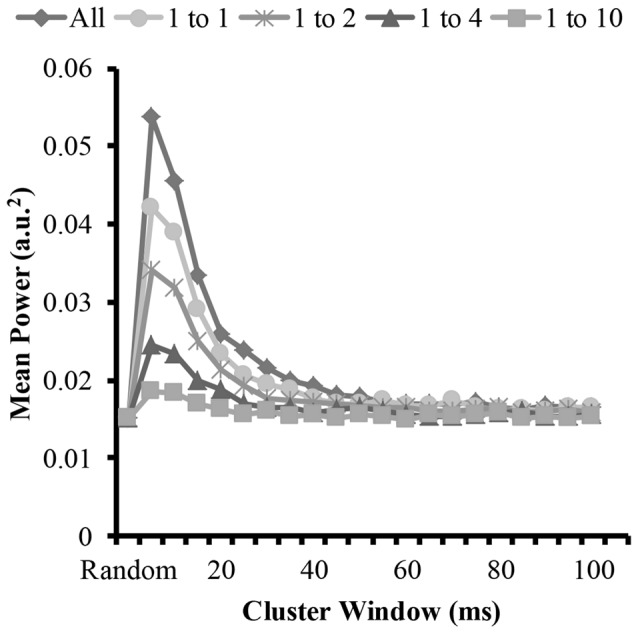
Mean power of each modeled EMG signal from the time-frequency analysis as a function of cluster-duration window size for a representative iteration. The diamond represents an EMG signal with all clustered MUAP. The circles represent the mean power of an EMG signal with a 1:1 ratio of clustered to randomly distributed MUAP. The asterisks represents the mean power of an EMG signal with a 1:2 ratio of clustered to randomly distributed MUAP. The triangles represent the mean power of an EMG signal with a 1:4 ratio of clustered to randomly distributed MUAP. The squares represent the mean power of an EMG signal with a 1:10 ratio of clustered to randomly distributed MUAP.

To answer the other part of the first and second hypothesis that clustering with varying ratios changes the EMG amplitude, we measured the amplitude of the modeled EMG signals. The time-frequency analysis revealed similar patterns to the mean frequency. Figure 6 shows the mean EMG power from the wavelet analysis for the different cluster durations for each cluster ratio. The analysis revealed that when the cluster duration was between 5 ms and 25 ms there was an increase in mean EMG power with the EMG created from the clustered MUAP. The analysis revealed that the increase in EMG power was the greatest when there was a large proportion of clustered MUAP to randomly distributed MUAP (i.e., ratio 1:1), while this effect was less pronounced when there was a smaller proportion of clustered MUAP to randomly distributed MUAP (i.e., ratio of 1:10).

The statistical analysis of the mean EMG power as a function of cluster ratio and cluster duration revealed that there was a significant interaction effect (*F*_(35.8,3541.3)_ = 6914.8, *p* < 0.001). From the significant interaction effect, the important comparisons are the differences in mean EMG power between the EMG signal created from different cluster ratios for a given cluster duration (e.g., comparison across five signals with a cluster duration of 10 ms) as well as the comparison of the mean EMG power of the EMG-rand signal in comparison to an EMG signal containing clustered MUAP (e.g., 1:1 cluster ratio) for a given cluster duration (e.g., 10 ms). For the comparisons across the different cluster ratios, every comparison was significantly different (*p* < 0.001). For the comparisons of the EMG-rand to the clustered EMG signal, the mean EMG power was significantly different for each clustered EMG signal in comparison to the EMG-rand signal (*p* < 0.001).

It could also be that the duration of the MUAP could also influence the results of our model. We tested this hypothesis by changing the duration of the MUAP up to 4× the duration of the MUAP in Figure 1. The results of this change in MUAP duration were very similar to the results presented above. For example, the power spectra shapes were very similar and in terms of percentage change, the mean frequency results were similar.

## Discussion

The present study examined the effect of MUAP clustering on features of an EMG power spectrum. A model was designed that systematically changed the sequence of MU firing. Specifically, the time window size that MUs were constrained to fire (i.e., cluster-duration) and the ratio of “clustered” MUAP to randomly distributed MUAP was altered. The results of the model indicated that changing these parameters caused substantial changes in the resulting EMG signal. MUAP clustering created a remarkable shift of the power spectrum’s mean frequency and altered the structure and shape of the power spectrum. Further, clustering of MUAP caused a substantial increase in the mean amplitude of the EMG power as a result of a time-frequency analysis. We believe that the modeled sequence of muscle activation during a simulated motor task has implications for the activation of muscles and the corresponding changes in EMG observed during fatiguing and clinical (e.g., tremor) conditions. In summary, our theoretical model shows that MUAP clustering greatly impacts an EMG and matches experimental work; we suggest that this phenomenon must be considered for future interpretation of EMG research.

### Mean Frequency

In support of hypothesis 1, our model indicated that MUAP clustering modified metrics of the power spectrum. Precisely, the mean frequency of an EMG created from “clustered” MUAP shifted to lower frequencies in relation to a model EMG from randomly distributed MUAP. The model indicated that when the cluster-duration was set to 10 ms and the EMG was comprised of only clustered MUAP, there was the largest downward shift in the mean frequency of the EMG from ~82 Hz to ~53 Hz (Figure 5; bottom line, diamonds). A downward shift in the EMG power spectrum has been seen experimentally elsewhere in the literature during an isometric vs. dynamic contraction (Merlo et al., 2005), eccentric contractions (Linnamo et al., 2002), submaximal vs. maximal effort contractions in certain muscles (Pincivero et al., 2002), fatiguing dynamic contractions (Komi and Tesch, 1979; Smale et al., 2016), and purely isometric fatiguing contractions (Bigland-Ritchie et al., 1981; Mills, 1982; Kuorinka, 1988; Krogh-Lund and Jørgensen, 1991). Further, the magnitude of the downward shift of the mean frequency was dependent on the size of the cluster duration window and the ratio of clustered to randomly distributed MUAP. When the cluster duration window increases, the mean and median frequency also shifts to lower values. This downward shift, however, is non-linear such that the magnitude of the downward shift for larger cluster durations (15–40 ms) is less than the downward shift observed with the 10 ms cluster duration window.

In support of hypothesis 2, the effects of altering the cluster ratio were similar such that less “clustered” MUAP resulted in a less substantial downward shift in the metrics of the power spectrum. The lowering value of the mean frequency of the power spectrum are typically attributed to a widening of the MUAP due to slowing of the conduction velocity (Eberstein and Beattie, 1985; Arendt-Nielsen et al., 1989). Dimitrova and Dimitrov (2003) developed an extensive model to explain the effects of fatigue on an EMG and indicated that MUAP width contributes to the lowering in mean and median frequency seen in fatigue, but that MUAP width does not fully explain this reduction. Previous modeling work studied the effects of short-term synchronization on EMG spectra and indicated that synchronization also contributes to this reduction in mean frequency (Yao et al., 2000). We suggest that, in addition to MUAP width and MU synchronization, MUAP clustering also contributes to the change in these metric of the power spectrum during fatiguing contraction and that only a small degree of clustering is required to change EMG spectra.

### EMG Amplitude

The results of the time-frequency analysis (i.e., wavelet transformation) indicated that there was an increase in EMG amplitude characteristics when the clustering windows were in the shorter range (i.e., 5–25 ms) in support of hypothesis 1. Compared to the longer clustering window sizes, this shorter time window resulted in a 100% increase the mean amplitude of the EMG signal. Further, and in support of hypothesis 2, all cluster ratios caused an increase in EMG amplitude. This change in the EMG signal has been observed frequently during muscle fatigue and dynamic tasks. During sustained isometric contractions, there is anywhere between a 50% and 100% increase in EMG amplitude from a baseline recording when a person holds an isometric contraction to volitional exhaustion (Petrofsky et al., 1982; Fuglevand et al., 1993b; Dideriksen et al., 2010). We suggest that given our model results that this increase in EMG amplitude could be attributed to MUAP clustering. This finding is similar to the modeling results of Yao et al. (2000) that showed that short-term synchronization (MU within a 5 ms window) increases EMG power similarly with a moderate and high level of synchrony. We extend these model findings and indicate that MU clustering with window sizes up to 25 ms also creates increases in EMG power. Further, during dynamic tasks such as running, it is frequently observed that the EMG signal from muscles such as the gastrocnemius are higher during running vs. isometric maximal voluntary contractions (MVCs; Lucas-Cuevas et al., 2016). Given that during running the muscle is constrained to fire in a short time period, the motor units are likely clustered. We speculate that the increased EMG activity above an MVC value during running is due to this clustering of motor units. In sum, motor unit action potential clustering creates an increase in the amplitude of the EMG signal and this increase has been observed experimentally during fatiguing and dynamic tasks and matches previously modeled EMG signals.

### Power Spectrum Shape

MUAP clustering modulated the shape and structure of the power spectrum. In Figure 3C, a power spectrum computed from randomly distributed MUAPs has a relatively normal distribution with only one peak. This shape of the power spectrum is similar to the power spectrum recorded from a surface EMG signal experimentally (e.g., Bigland-Ritchie et al., 1981; Hägg, 1992). By modifying the cluster-duration size, this shape of the power spectrum drastically changes. A very short cluster-duration window size (i.e., 10 ms) creates a downward shift in the peak of the power spectrum to lower frequencies and creates a positively skewed distribution (Figure 4, third line from the top). This change in the power spectrum shape from our model mimics the changes in the shape of the power spectrum observed frequently in the literature during fatiguing conditions (Bigland-Ritchie et al., 1981; Mills, 1982; Kuorinka, 1988; Krogh-Lund and Jørgensen, 1991). Having a larger cluster-duration size (i.e., 15–40 ms), however, changes the shape of the power spectrum differently. A larger cluster-duration produces a power spectrum with a bimodal (Figure 4, 5th line from top) or even a multimodal distribution (Figure 4, lines 6–9 from top) with a peak occurring around 40 Hz. This type of power spectral shape change has been reported less frequently, although it has been observed in the literature during fatiguing contractions and clinical conditions such as a tremor (van Boxtel and Schomaker, 1984; Rossi et al., 1996; Chen et al., 1997; von Tscharner et al., 2011, 2014). In general, these changes in the EMG power spectrum appear to be focused mainly on the low frequency component and it is likely that our modeled activation pattern of the muscle drive these changes as suggested elsewhere (Pan et al., 1989). Overall, it seems apparent that changing the cluster-duration creates two distinct changes in the shape and structure of the power spectrum and these distinct shapes are observed experimentally.

### Functional Relevance

Piper (1912) indicated that there are bursts of activity in the EMG during sustained contractions and that these bursts may indicate a control signal from the central nervous system. These bursts of activity are typically in the order of 20–60 Hz (Piper, 1912; Fex and Krakau, 1958; Brown et al., 1998). From our model, constraining the MUAP to arrive together within 10 ms caused the mean frequency to shift from ~50 Hz to ~80 Hz. This lowered value of the mean frequency is observed during isometric fatiguing contraction could also be due to the clustered activation of the muscle at lower frequencies (i.e., 20–60 Hz), where MUAP preferentially arrive at the maxima of the signal. In support of this statement, it has been shown recently that during fatiguing isometric contractions, the coherence of motor units increases in the delta (1–4 Hz), alpha (8–12 Hz), but also the beta band (15–30 Hz). It could be that this coherence increase could be driven by more synchronous activity of motor units and thus, a result of MUAP clustering. This activation pattern of clustering may also be evident in maximal effort isometric tasks. Merlo et al. (2005) had individuals perform maximal and ramped isometric contractions while squatting and indicated that the mean frequency changed to a lower frequency (i.e., 55 Hz) during the maximal effort task. This change in the mean frequency matched the results from our MUAP clustering model. We suggest that during the maximal effort squat, participants clustered MUAP during this brief period to produce the large force requirements of the task. This clustering of MU would support the idea that synchronization, or clustering, is an aspect of muscle control that can be trained to produce more force. Previous work (Semmler et al., 2004; Fling et al., 2009) has shown that the level of synchronization is increased in strength trained individuals. It could be that clustering of MU is a method to increase force depending on the task requirement. In sum, our model suggests that MUAP clustering has functional relevance for both maximal effort and fatiguing isometric contractions and we can conclude that this model is similar to what is observed in reality.

It has been suggested that this clustered activation pattern emerges not only in sustained muscle contractions, but also during dynamic tasks. For example, during running, the stance phase is very short (i.e., 200–400 ms depending on the speed) and the muscle is only required to be active for this short period of time. Theoretically for this dynamic task, MUAP are constrained to fire together for a precise period or simply “cluster” together. In Maurer et al. (2013), participants ran at different speeds and the results indicated that a rhythm emerged in the EMG of a muscle controlling the lower limb. We suggest that these bursts in muscle activity during the running task were due to MUAP clustering that was controlled by the CNS during this task. It has previously been suggested that during dynamic and isometric tasks a muscle rhythm may reflect a control single originating in motor cortex (Hagbarth et al., 1983; Salenius et al., 1997; Brown et al., 1998; Mima et al., 2001; Funk and Epstein, 2004). To further support this suggestion, Clark et al. (2013) also revealed synchronous activation of the triceps surae muscle group in the Piper band width (20–60 Hz) that was dependent on the tasks with varying corticospinal tract involvement (Clark et al., 2013). Further, we have found similar results of a rhythmic activation of a muscle using non-linearly scaled wavelets (von Tscharner et al., 2003). We suggest that the bursts of muscle activity that appear in the EMG signal are due to clustering of motor units and future research should determine how the control signal from the CNS is generated to produce this rhythmic activation of the muscle.

### Synchronization, Firing Rate and Clustering

Previous research has examined how the firing rate of the motor units influences power spectrum (Farina et al., 2004a). Our clustering model is not simply based on the firing rate of motor units, but instead is structured such that motor units are constrained to fire within certain cluster window sizes (i.e., 5–100 ms) and is much different from the results presented by Farina et al. (2004a), the model created by Yao et al. (2000) and long-term synchronization (De Luca et al., 1993). Short-term synchronization seems to play an important role in the resulting EMG signal. Our results add to the literature, as we have shown that motor units that are constrained fire within discrete windows largely influence the EMG signal and that this clustered activation of motor units may be an important aspect of producing the required force output for a given task. We believe our evidence shows a link between the simulation results and a physiological interpretation. For instance, for humans to produce large amounts of force or hold a contraction until volitional exhaustion, based on Sherrington’s final common path, the only way to increase force it to increase the recruitment of motor units and increase the firing rate of motor units. These two increases could lead to motor units to become “clustered” by chance of more motor units being active. We speculate that this type of clustered activation is not by chance, but may be driven by a special strategy of the neural system during high force production tasks or to cope with motor unit fatigue. For example, clustering may be necessary to cope with changing force loads in a task or a way to produce larger, intermittent burst of force in the event of muscle fatigue. Further experimental data combined with models of motor unit fatigue (Potvin and Fuglevand, 2017) may help determine the possibility of this pattern of activation during a fatiguing task. We believe that this model provides novel insight into changes caused by altering clustering of the potential firing patterns during muscle fatigue, dynamic tasks and maximal isometric efforts–an effect that to date has largely been overlooked.

### Limitations and Future Directions

We understand that the model presented in this article is a simplified and we have only presented a model and how this model relates to experimental work. For the model, we intentionally did not include more complexity because we wanted to produce a model with the minimal amount of features to explain the phenomenon of MUAP clustering. In the model, we also kept the number of active motor units constant and a random distribution of MUAP, and did not include motor unit recruitment patterns because once again, we wanted to isolate the effects of MUAP clustering. Further work should build on the model and include specific (e.g., periodic) recruitment patterns, firing rate, volume conductor effects, and number of motor units found in different muscles to determine how MUAP clustering may be muscle specific. Although the model is simplified, we believe that it is important to use a model such as the one presented here to understand, analyze, and interpret EMG signals, particularly during dynamic tasks.

## Conclusion

We presented a modified classical model of an EMG using a “clustered” MU firing sequence. MUAP clustering creates substantial changes in the shape and structure of the power spectrum. In dynamic tasks, where there are short intervals of muscle activity, MUAP clustering, theoretically, is likely the most logical method of neuromuscular control. The results of our model indicate that in the interpretation of power spectra during dynamic tasks, the possible modifications of the power spectra due to clustering should be considered. The general measure for fatigue is the lowering of the mean/median frequency and we suggest that the lowering of the mean/median can also be driven by MUAP clustering. Interestingly, the lowering of these metrics, because of clustering, could be a measure of the activation patterns of muscles and therefore, this effect must clearly be separated from peripheral effects in future research. Our model indicates that the rhythmic activation of a muscle is due to clustering of motor units and this clustering dramatically alters EMG spectra and amplitude similar to what is seen in fatiguing and clinical conditions.

## Author Contributions

MJA and VT were responsible for the conception of the idea and design of the model. BMN, MJA and VT were responsible for the interpretation of the work, drafting and revising the manuscript and final approval of the manuscript.

## Conflict of Interest Statement

The authors declare that the research was conducted in the absence of any commercial or financial relationships that could be construed as a potential conflict of interest.
